# ANAC042 Regulates the Biosynthesis of Conserved- and Lineage-Specific Phytoalexins in Arabidopsis

**DOI:** 10.3390/ijms26083683

**Published:** 2025-04-13

**Authors:** Ivan Monsalvo, Leonardo Parasecolo, Sarah Pullano, Jie Lin, Aida Shahabi, Melissa Ly, Hyejung Kwon, Khushi Mathur, Karl Angelo M. Rodrillo, Demian R. Ifa, Nik Kovinich

**Affiliations:** 1 Department of Biology, Faculty of Science, York University, 4700 Keele St., Toronto, ON M3J 1P3, Canada; imm1802@my.yorku.ca (I.M.); pullans@my.yorku.ca (S.P.); lj0215@yorku.ca (J.L.); aida2022@my.yorku.ca (A.S.); mthanhly@my.yorku.ca (M.L.); hkwon96@my.yorku.ca (H.K.); khushi31@my.yorku.ca (K.M.); 2 Department of Chemistry, Faculty of Science, York University, 4700 Keele St., Toronto, ON M3J 1P3, Canada; leopara8@yorku.ca (L.P.); verbal59@yorku.ca (K.A.M.R.); ifadr@yorku.ca (D.R.I.)

**Keywords:** *Arabidopsis thaliana*, transcription factor, pleiotropy, phytoalexin, basal immunity

## Abstract

Phytoalexins are specialized metabolites that are synthesized by plants in response to pathogens. A paradigm in transcription factor (TF) biology is that conserved TFs have dedicated roles across plant lineages in regulating specific branches of specialized metabolism. However, the Arabidopsis (*Arabidopsis thaliana*) NAC family TF ANAC042 (a.k.a. JUNGBRUNNEN1 or JUB1) regulates the synthesis of camalexin, a Trp-derived phytoalexin specifically produced by several *Brassicaceae* species, whereas its homolog in soybean (*Glycine max*) regulates the synthesis of glyceollins, which are Phe-derived phytoalexins specific to soybean. The question addressed by this research is whether ANAC042 broadly regulates phytoalexin biosynthetic pathways in Arabidopsis. Using a novel matrix-assisted laser desorption ionization high-resolution mass spectrometry (MALDI-HRMS) method, we found that the Arabidopsis loss-of-function mutant *anac042–1* elicited with bacterial flagellin (Flg22) is deficient in lineage-specific Trp- and conserved Phe-derived phytoalexins—namely camalexin and 4-hydroxyindole-3-carbonyl nitrile (4OH-ICN), and pathogen-inducible monolignols and scopoletin, respectively. Overexpressing *ANAC042* in the *anac042-1* mutant restored or exceeded wildtype amounts of the metabolites. The expression of phytoalexin biosynthetic genes in mutant and overexpression lines mirrored the accumulation of metabolites. Yeast-one hybrid and promoter-reporter assays in *Nicotiana benthamiana* found that the ANAC042 protein directly binds and activates the promoters of *CYP71B15*, *CYP71A12*, and *PAL1* genes for the synthesis of camalexin, 4OH-ICN, and pathogen-inducible monolignol/scopoletin, respectively. Our results demonstrate that ANAC042 regulates conserved and lineage-specific phytoalexin pathways in Arabidopsis. The latter suggests that it is an opportunistic TF that has coopted lineage-specific genes into phytoalexin metabolism, thus providing an exception to the current paradigm.

## 1. Introduction

As sedentary organisms, plants rely on genetic reprogramming to activate the expression of defense traits in order to protect themselves against biotic threats. Upon recognizing conserved pathogen-associated molecular patterns (PAMPs), such as the 22-amino acid fragment of the bacterial peptide flagellin (Flg22), signaling cascades ensue that ultimately stimulate the expression of transcription factors (TFs) that directly activate the transcription of genes for expression of defense traits [1,2,3,4]. An important “early” defense trait in plant immunity is the synthesis of phytoalexins. Phytoalexins are defined as plant-specialized metabolites that are biosynthesized de novo in response to pathogens. The role of phytoalexins in mediating resistance to microbial pathogens was first established using gene mutants of the model plant *Arabidopsis thaliana* (Arabidopsis). Camalexin, a.k.a. 3-thiazol-2′-yl-indole, was characterized as the major phytoalexin produced by Arabidopsis in response to *Pseudomonas syringae pv syringae* [5]. Genetic screens of Arabidopsis T-DNA mutant collections identified phytoalexin deficient (*PAD*) mutants that have reduced camalexin amounts. These mutants demonstrated that camalexin deficiency results in reduced resistance to various microbial pathogens [6,7,8,9]. Genetic mapping of *pad* mutants identified genes for the signaling and biosynthesis of camalexin, rendering camalexin a hallmark defense trait for the dissection of molecular components of basal immunity [10,11,12]. Basal immunity signaling components identified using camalexin levels as a proxy include pathogen recognition receptors (PRR) [13], hormone signaling proteins [14,15], mitogen-activated protein kinases (MPK3/MPK6) [16,17,18], calcium-dependent protein kinases (CDPKs) [19,20,21], and TFs that directly activate the expression of camalexin genes [22,23].

Collectively, plants synthesize phytoalexins from nearly every branch of specialized metabolism [24]. Some of these pathways are broadly conserved in vascular plants, whereas others are restricted to individual plant lineages or species. The conserved Phe-derived phytoalexins scopoletin and pathogen-inducible monolignols are biosynthesized broadly by dicots, monocots, and magnoliids [25]. Stilbenes have a narrower distribution, being found in grapevine (*Vitis vinifera*), peanut (*Arachis hypogaea*), and various pulses. In contrast, species-specific Phe-derived phytoalexins include pisatin from peas (*Pisum sativum*), medicarpin from alfalfa (*Medicago sativa*), sakuranetin from rice (*Oryza sativa*), and the glyceollins from soybean (*Glycine max*).

Lineage-specific phytoalexins derived from Trp include the camalexin and the brassinins from the *Brassicaceae*, oxoglaucines from *Magnoliaceae*, DIMBOA from *Zea maize*, and the avenanthramides from oat (*Avena sativa*). 4-hydroxyindole-3-carbonyl nitrile (4OH-ICN) is a Trp-derived indole alkaloid that has been found exclusively in Arabidopsis. A recent review found that Arabidopsis actually synthesizes at least 12 phytoalexins [25]. While much is known about the transcriptional regulation of camalexin biosynthesis, studying this pathway in isolation has informed little on whether regulators may be shared amongst conserved or other lineage-specific phytoalexin pathways.

In Arabidopsis, previous studies have shown that the phytoalexins camalexin, 4OH-ICN, lignin, and scopoletin contribute to resistance against *P. syringae* and *Botrytis cinerea* [26,27,28,29]. Lignin and scopoletin are also involved in defense against *Fusarium oxysporum* [30,31,32], while camalexin and 4OH-ICN are associated with resistance to *Alternaria brassicicola* [25,33,34]. These observations suggest that conserved phytoalexins (lignin, scopoletin) may confer broad-spectrum resistance, whereas lineage-specific phytoalexins (camalexin, 4OH-ICN) may target host-specific pathogens such as *A. brassicicola*. However, the defensive role(s) of each phytoalexin in relation to the pathogen range remain to be fully characterized. TFs that regulate phytoalexin biosynthesis have been identified in numerous plant species from gene families, including NAM, ATAF1/2, CUC (NAC), myeloblastosis related (MYB), WRKYGQK motif (WRKY), basic helix-loop-helix (bHLH), and APETALA2/ethylene-responsive factor (AP2/ERF). Those that regulate camalexin biosynthetic genes are WRKY, MYB, ERF, and NAC family proteins [35]. WRKY33 directly regulates the transcription of early-stage Trp-derived phytoalexin gene *CYP79B2/3*, the 4OH-ICN gene *CYP71A12*, and the camalexin gene *CYP71B15/PAD3* [23,33,36]. WRKY33 loss-of-function mutants exhibit a nearly complete reduction in camalexin amounts [37,38], suggesting a lack of functional redundancy. Loss-of-function mutants of the R2R3 MYB genes *MYB51* and *MYB122* exhibit a little reduction in camalexin amounts; however, the *myb51 myb122* double mutant has a major reduction [39]. MYB51 and MYB122 proteins directly bind the promoters of *CYP79B2* and *CYP79B3* but do not regulate *CYP71A13* or *CYP71B15/PAD3* [39]. The AP2/ERF family protein ERF72 directly binds and activates the promoters of *WRKY33*, *CYP71A13*, and *CYP71B15/PAD3* [18]. ERF1 also directly activates the expression of *CYP71A13* and *CYP71B15/PAD3* and forms a transcriptional complex with WRKY33 that enhances WRKY33′s transactivation of camalexin gene promoters [14].

T-DNA insertion mutants of the NAM, ATAF1/2, and CUC2 (NAC) family TF *ANAC042* are deficient in camalexin amounts upon elicitation with Flg22, *Alternaria brassicicola*, AgNO_3_, or the herbicide acifluorfen [22]. The mutants exhibit reduced expression of camalexin biosynthetic genes *CYP71A12*, *CYP71A13*, and *CYP71B15*/*PAD3*, suggesting that the ANAC042 protein directly or indirectly regulates camalexin biosynthesis. In the absence of elicitation, overexpressing *ANAC042* (a.k.a. *JUNGBRUNNEN1* or *JUB1*) upregulates the expression of reactive oxygen species (ROS)-responsive genes and enhances tolerance to various abiotic stresses [40]. Thus, whether ANAC042 has a general role in ROS signaling or directly regulates phytoalexin biosynthetic genes remains unknown.

The TFs that regulate the expression of Phe-derived phytoalexins have been characterized in several plant species. In rice, the bHLHs OsMYC2, OsMYC2-like protein 1/2 (OsMYL1/2), and the 1R MYB TF OsMYB1R, directly bind and regulate the promoter of the sakuranetin biosynthetic gene *OsNOMT* [41,42]. In Arabidopsis, loss-of-function mutants of the R2R3 MYB family gene *MYB15* exhibit reduced amounts of pathogen-inducible lignin and scopoletin [28]. The MYB15 protein directly binds and activates the promoters of early Phe-derived phytoalexin biosynthetic genes *PAL*, *C4H*, and *4CL*, the monolignol gene *COMT*, and the scopoletin gene *F6′H1* [28]. However, in soybean, the *MYB15* homolog *GmMYB29A2* encodes a protein that directly binds and activates the promoters of glyceollin biosynthetic genes *GmIFS2* and *GmG4DT* [43]. Similarly, the soybean homolog of *ANAC042*, namely *GmNAC42–1*, directly regulates glyceollin biosynthetic genes [44]. This raises the question of whether phytoalexin TFs such as ANAC042 broadly regulate conserved and lineage-specific phytoalexin pathways.

To test this hypothesis, we used a matrix-assisted laser desorption ionization high-resolution mass spectrometry method to determine whether ANAC042 regulates both Phe- and Trp-derived phytoalexin biosynthetic pathways of Arabidopsis. Using a loss-of-function mutant, gene overexpressors, and DNA-binding and promoter-reporter assays, we demonstrate that ANAC042 is a direct regulator of both conserved Phe and lineage-specific Trp phytoalexin pathway genes. Our results inform on the plasticity of phytoalexin gene regulation, identifying ANAC042 as a key regulator of conserved and lineage-specific phytoalexin pathways in Arabidopsis. The results begin to explain how conserved TFs have evolved to regulate the distinct biochemical defenses of different plant lineages. They suggest an adaptive mechanism whereby conserved TFs opportunistically coopt lineage-specific defenses in response to selective pressures to actively shape metabolic innovation. This provides an exception to the current paradigm that conserved TFs have dedicated roles across plant lineages in regulating specific branches of specialized metabolism.

## 2. Results

### 2.1. Arabidopsis Loss-of-Function Mutant anac042-1 Is Deficient in Phe- and Trp-Derived Phytoalexins

**Figure 1:**
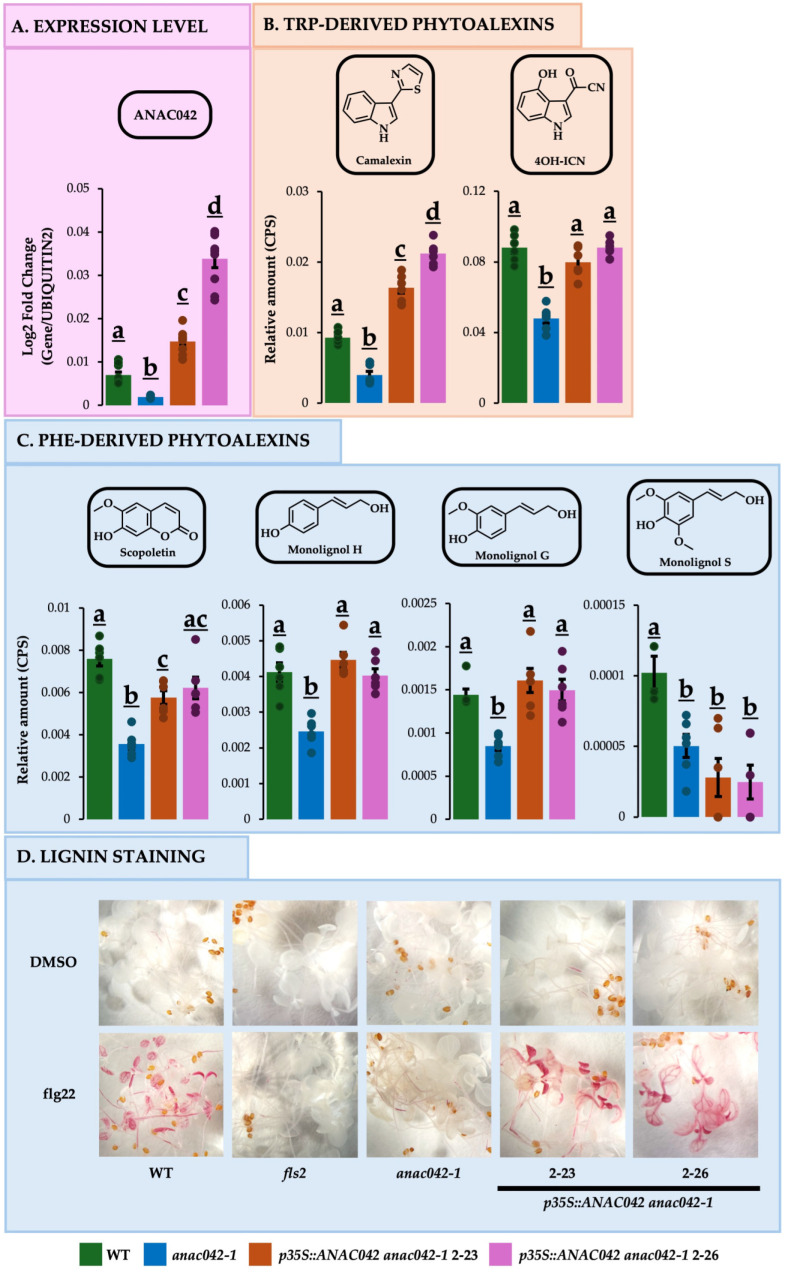
Phytoalexin metabolite profiles and lignin staining of *ANAC042* mutant and gene overexpression lines. (**A**) *ANAC042* expression levels and (**B**,**C**) phytoalexin profiles of seedling extracts quantified by matrix-assisted laser desorption ionization high-resolution mass spectrometry (MAL-DI-HRMS) relative to daidzein (internal standard) and qRT-PCR relative to *UBIQUITIN2*, respectively. Ten-day-old seedlings were elicited with Flg22, and metabolites were measured at 12 h post-elicitation. The significance test was performed by single factor ANOVA, Tukey post hoc test, which is indicated by different letters (*p* < 0.01). Error bars represent SE (*n* ≥ 3). For a list of statistical values, see Supplementary Table S1. (**D**) Lignin staining with phloroglucinol-HCl under 16× magnification. Seven-day-old seedlings were elicited with flg22 and stained 48 h post-elicitation.

*ANAC042* has been implicated in regulating camalexin biosynthesis in Arabidopsis [22], whereas its homolog in soybean, *GmNAC42-1*, regulates the biosynthesis of glyceollins [44]. Thus, we hypothesized that NAC42-type TFs could have broad roles in regulating diverse phytoalexin pathways in plants. To test this hypothesis in Arabidopsis, we compared the phytoalexin profiles of flg22-treated loss-of-function mutant *anac042-1* to the wildtype. We confirmed that *ANAC042* gene expression levels and camalexin metabolite levels are reduced in *anac042-1* (Figure 1A,B). We also observed reduced amounts of the Trp-derived phytoalexin 4OH-ICN (Figure 1B).

Notably, the levels of the Phe-derived phytoalexins scopoletin and pathogen-inducible monolignols were also decreased in *anac042-1* (Figure 1C). Furthermore, *anac042-1* failed to undergo lignification upon flg22 treatment, unlike the WT (Figure 1D). These results suggest new roles of ANAC042 in regulating diverse Trp- and Phe-derived phytoalexin pathways in Arabidopsis.

### 2.2. Overexpressing ANAC042 in anac042-1 Background Restored or Exceeded Wildtype Amounts of Phe- and Trp-DERIVED Phytoalexins

Previously, it was reported that expressing *ANAC042* with a 1.5 kb fragment of its native promoter in the *anac042-1* background partially complemented camalexin biosynthesis [22]. To test the putative function of ANAC042 in regulating phytoalexin biosynthesis, we transformed *ana042-1* with the *ANAC042* coding sequence expressed using the viral *35S* promoter. The transgenic lines *p35S::ANAC042-*2-23 and *p35S::ANAC042-*2*ANAC042-*2*-*26 overexpressed *ANAC042* 2.1- and 4.9-fold, respectively (Figure 1A), and exhibited higher levels of camalexin than *anac042-1* and the WT (Figure 1B). Further, 4OH-ICN levels were restored to those of the WT (Figure 1B).

The amount of monolignols H and G were restored to WT levels in both overexpressing lines (Figure 1C). However, monolignol S amounts were the same as *anac042-1*, suggesting the preferential activation of monolignols H and G. Scopoletin levels were partially and fully complemented in 2–23 and 2–26 (Figure 1C), respectively. Moreover, the two overexpression lines recovered the lignification phenotype lost in the loss-of-function mutant *anac042-1* (Figure 1D). Taken together with loss-of-function results, gene overexpression demonstrated that ANAC042 positively regulates the biosynthesis of Trp- and Phe-derived phytoalexins.

### 2.3. Overexpressing ANAC042 in anac042-1 Background Restored or Exceeded Wildtype Levels of Phe and Trp Phytoalexin Gene Expressions

**Figure 2:**
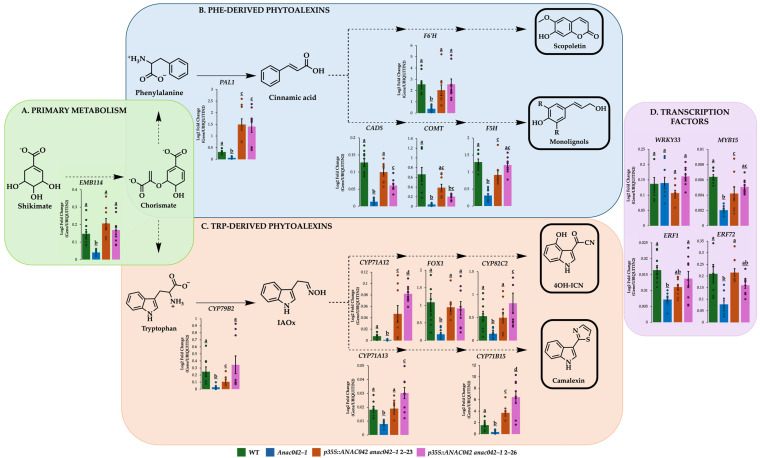
Phytoalexin gene expression profiles of *ANAC042* mutant and gene overexpression lines. (**A**) Primary metabolism gene *EMB114*; (**B**) Phe pathway phytoalexin genes; (**C**) Trp pathway phytoalexin genes; and (**D**) phytoalexin TFs. Gene expression of flg22-treated seedlings was measured by quantitative reverse transcription polymerase chain reaction (qRT-PCR) relative to *ACTIN2*. The significance test was performed by single factor ANOVA, Tukey post hoc test, which is indicated by different letters (*p* < 0.01). Error bars represent SE (*n* ≥ 3). For a list of statistical values, see Supplementary Table S2. *EMB1144*, chorismate synthase; *PAL1*, phenylalanine ammonia–lyase 1; *CAD5*, cinnamyl alcohol dehydrogenase 5; *COMT*, caffeic acid O–methyltransferase; *F5H*, ferulate–5–hydroxylase; *F6′H*, feruloyl–CoA 6′–hydroxylase; *CYP79B2*, cytochrome P450 79B2; *CYP71A12*, cytochrome P450 71A12; *CYP71A13*, cytochrome P450 71A13; *CYP71B15*, cytochrome P450 71B15; *FOX1*, 2-hydroxy-2-(1H-indol-3-yl)acetonitrile oxidase; *CYP82C2*, cytochrome P450 82C2.

To determine whether ANAC042 affects phytoalexin metabolite levels through the expression of their biosynthetic genes, we measured biosynthetic gene expressions using qRT-PCR. *EMB114*, a primary metabolism gene involved in Trp and Phe biosynthesis, was downregulated in flg22-treated *anac042-1* compared to WT. Its expression levels were restored to WT levels in both *p35S::ANAC042* overexpression lines (Figure 2A).

Phe-derived phytoalexin biosynthetic genes were downregulated in flg22-treated *anac042-1* compared to WT, including *PAL1*, the scopoletin gene *F6′H*, and the monolignol genes *CAD5*, *COMT*, and *F5H* (Figure 2B). In *ANAC042* overexpression lines, all gene expressions were fully or partially restored to WT levels and *PAL1* was upregulated by 4.5- 4.8-fold (Figure 2B).

Trp-derived phytoalexin biosynthetic genes were downregulated in flg22-treated *anac042-1* compared to WT, including camalexin genes *CYP79B2*, *CYP71A13*, and *CYP71B15*, and 4OH-ICN genes *CYP71A12*, *FOX1*, and *CYP82C2* (Figure 2C). In *p35S::ANAC042-2-23* and *2-26*, *ANAC042* overexpression restored the expression of *CYP79B2*, *CYP71A13*, *FOX1*, and *CYP82C2*, while upregulated *CYP71A12* (6- and 10.6-fold) and *CYP71B15* (2.4- and 4.2-fold), respectively (Figure 2C).

Phytoalexin TFs have been reported to regulate the expression of other phytoalexin TFs in addition to their biosynthetic genes [14]. To determine whether ANAC042 also regulated the expression of phytoalexin TF genes, we measured TF gene expressions using qRT-PCR. The expression of *WRKY33*, a Trp-derived phytoalexin regulator, remained unchanged across *anac042-1* and the *ANAC042* overexpression lines. However, camalexin regulators *ERF1* and *ERF72* were downregulated 2.3- and 2.7-fold in *anac042-1* and restored to WT levels in the overexpressors (Figure 2D). Similarly, the Phe-derived phytoalexin regulator *MYB15* was downregulated 3.1-fold in *anac042-1*, while its expression was partially or fully restored in the *ANAC042* overexpression lines (Figure 2D). These results demonstrate that *ANAC042* is a positive regulator of both Phe- and Trp-derived phytoalexin biosynthetic genes.

### 2.4. ANAC042 Directly Activates the Expression of Scopoletin, Monolignol, 4OH-ICN, and Camalexin Biosynthetic Genes

**Figure 3:**
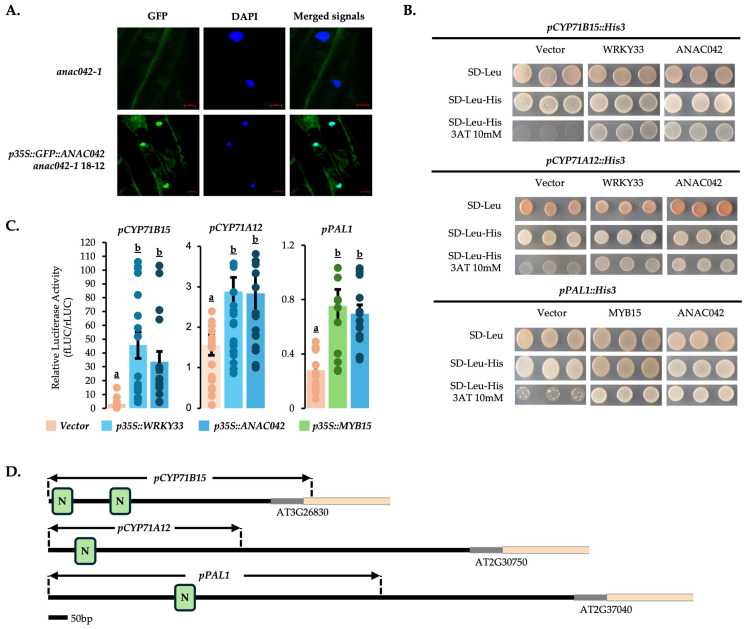
Characterization of the subcellular localization and protein-DNA interactions (PDIs) of ANAC042 protein. (**A**) Fluorescence microscopy of *p35S::GFP::ANAC042*-18*-*12. Seedlings from *anac042-1* were used as negative control. DAPI (6 µg/mL) images indicate nuclear staining. Bars in red represent 5 µm. (**B**) Y1H analysis of strain YM4271 transformed with *ANAC042-Gal4AD*, *WRKY33-Gal4AD*, or *MYB15-Gal4AD*, and *pCYP71B15::HIS3*, *pCYP71A12::HIS3,* or *pPAL1::HIS3*. SD-Leu plates were used as control for TF transformation; SD-Leu-His as control for TF and promoter transformation; and SD-Leu-His + 10 mM 3-aminotriazole (3AT) as indicators for positive PDIs. pDEST-GADT7 was used as the empty ‘Vector’ control. (**C**) Luciferase transactivation assay of transiently transformed *N. benthamiana* leaves with *p35S::ANAC042*, *p35S::WRKY33*, or *p35S::MYB15*, and *pCYP71B15::LUC*, *pCYP71A12::LUC*, or *pPAL1::LUC*. Luciferase activity measurements were performed on 48 h post-infiltrated leave lysates. p62GW was used as the empty ‘*Vector*’ control. The significance test was performed by single factor ANOVA, Tukey post hoc test, which is indicated by different letters (*p* < 0.01). Error bars represent SE (*n* ≥ 3). For a list of statistical values, see Supplementary Table S3. (**D**) Schematic diagram demonstrating promoter fragments of *CYP71B15*, *CYP71A12*, and *PAL1* used for yeast one-hybrid and luciferase transactivation assays. N-box elements with either 5′-GCCGT-3′ or 5′-ACGGC-3′ sequences (green boxes).

To confirm that ANAC042 exhibits nuclear localization, consistent with its putative role as a transcription factor [22], we expressed a translational fusion of the *ANAC042* coding sequence with an N-terminal green fluorescent protein (GFP) tag in the *anac042-1* mutant. Confocal microscopy confirmed nuclear localization of GFP-ANAC042 (Figure 3A). To determine whether the ANAC042 protein directly binds and regulates phytoalexin gene promoters, we conducted yeast one-hybrid and promoter-reporter assays, respectively.

Promoter sequence analysis revealed that *PAL1*, *CYP71A12*, and *CYP71B15*, which are phytoalexin genes that are differentially expressed in *ANAC042* mutant and overexpression lines (Figure 2B,C), each contain one to two ANAC042 recognition sites within 1.5 kb of their start codons (Figure 3D). To assess whether ANAC042 direct binds the promoter regions of those genes, we performed yeast one-hybrid (Y1H). First, we confirmed that WRKY33 interacts with the promoters of *CYP71A12* and *CYP71B15* [17,45] and that MYB15 interacts with the *PAL1* promoter [28] (Figure 3B). A translational fusion of the ANAC042 coding sequence with the Gal4 activation domain (AD) interacted with the promoters of 4-OH-ICN/camalexin genes *CYP71A12* and CYP71B15 (Figure 3B), as did the positive control WRKY33 [17,45]. ANAC042 also interacted with the pathogen-inducible monolignol/scopoletin gene *PAL1* (Figure 3B), as did the positive control MYB15 [28].

To determine whether ANAC042 can activate those promoters in planta, we conducted promoter-reporter (luciferase) assays in *Nicotiana benthamiana*. Similar to Y1H assays, WRKY33 and MYB15 were used as positive controls. Co-infiltration of *p35S::ANAC042* with *pPAL1::LUC*, *pCYP71A12::LUC*, or *pCYP71B15::LUC* resulted in similar levels of transactivation compared to the WRKY33 and MYB15 controls (Figure 3C). Together, these results establish that ANAC042 is a direct activator of Phe and Trp genes for phytoalexin biosynthesis.

## 3. Discussion

### 3.1. ANAC042 Is a Regulator of Diverse Phytoalexin Biosynthetic Pathways in Arabidopsis

**Figure 4:**
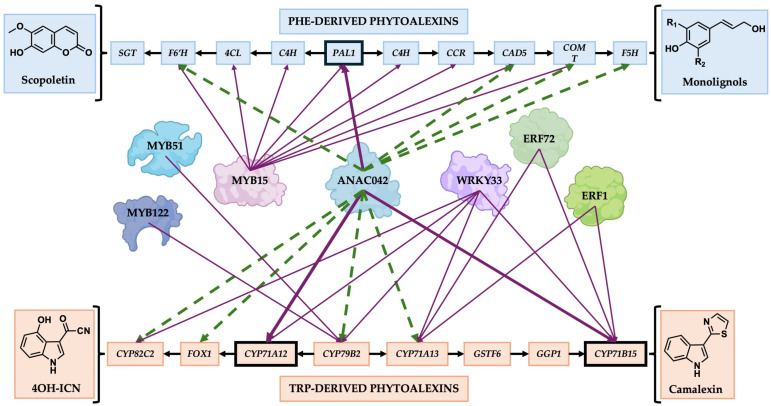
A schematic diagram of Phe- and Trp-derived phytoalexin biosynthetic genes in Arabidopsis and the transcription factors that regulate their expression. Black arrows (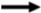) indicate the direction of genes involved in phytoalexin biosynthesis starting at *PAL1* (Phe-derived phytoalexins) and *CYP79B2* (Trp-derived phytoalexins); Purple arrows (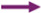) indicate direct regulation of the gene; Green dotted arrows (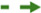) indicated regulation of the gene by qRT-PCR, but that it remains unknown whether the corresponding genes are regulated directly or indirectly. Gene names: *GSTF11*, *GLUTATHIONE S–TRANSFERASE F11*; *GGP1*, *Γ–GLUTAMYL PEPTIDASE 1*; *C4H*, *CINNAMIC ACID 4–HYDROXYLASE*; *4CL*, *4–COUMARATE–COENZYME A LIGASE*; *CCR*, *CINNAMOYL–COA REDUCTASE*; *SGT*, *SCOPOLETIN–GLUCOSYLTRANSFERASE*.

Arabidopsis synthesizes phytoalexin metabolites from both conserved and lineage-specific pathways [25]. Camalexin is a model phytoalexin elicited by stressors, such as acifluorfen (herbicidal compound), AgNO_3_ (ethylene inhibitor), flg22 (PAMP), and UV irradiation, yet it is synthesized specifically in only a few *Brassicaceae* species [22,33]. Thus, it remains questionable how much insight gained from the regulation of camalexin genes can be applied to other phytoalexin pathways. The TFs that positively regulate camalexin biosynthesis are MYB51, MYB122, WRKY33, ERF1, ERF72, and ANAC042 [14,18,22,25,39] (Figure 4). MYB51 and MYB122 proteins directly bind the promoters of *CYP79B2* and *CYP79B3*, which encode enzymes that convert Trp to indole-3-acetaldoxime (IAOx) (Figure 4). However, they do not directly regulate the promoters of *CYP71B15/PAD3* or *CYP71A12* for camalexin or 4OH-ICN biosynthesis, respectively [39]. WRKY33, ERF1, and ERF72 directly bind and activate the *CYP71B15/PAD3* promoter and thus are direct regulators of camalexin biosynthesis [14,18,19,45] (Figure 4). WRKY33 also regulates the biosynthesis of 4OH-ICN, a phytoalexin synthesized exclusively in Arabidopsis [33], demonstrating that it has a broader role than has been determined for other camalexin regulators. However, the role of ANAC042 has remained less clear. *anac042* loss-of-function mutants have reduced expression of camalexin metabolites and biosynthetic genes upon abiotic and biotic elicitation [22]. However, it has remained unknown whether ANAC042 directly binds and regulates camalexin biosynthetic genes or whether its regulation is indirect, for example, by regulating the expression of other camalexin TFs. We recently found that ANAC042′s homolog in the legume soybean regulates the synthesis of glyceollins, which are Phe-derived phytoalexins that are specific to soybean [44]. Our study raised the question of whether NAC42-type TFs have a broader role in regulating phytoalexin biosynthetic pathways in plants and thus whether information on camalexin gene regulation in Arabidopsis can be applied to other phytoalexins in other plants species [35].

In this study, we focused on clarifying the role of ANAC042 in Arabidopsis. Our experiments found that ANAC042 is a direct regulator of conserved phytoalexin pathways. Specifically, the loss-of-function mutant and gene overexpression lines demonstrated that ANAC042 positively regulates the biosynthesis of pathogen-inducible monolignols and scopoletin, which are Phe-derived phytoalexins that are biosynthesized broadly by dicots, monocots, and magnoliids [25]. Our results also showed that ANAC042 positively regulates the expression of camalexin and 4-hydroxyindole-3-carbonyl nitrile (4OH-ICN), which are specifically synthesized by a few species of *Brassicaceae*. Our DNA-binding and promoter-reporter assays suggest that ANAC042 does so by directly binding and activating the expression of conserved and lineage-specific biosynthetic genes, namely *PAL* and *CYP72A12/CYP71B15*, respectively. These findings are the first to identify a TF that regulates both conserved and lineage-specific phytoalexin pathways and that regulates phytoalexins that are derived from different amino acids. Thus, ANAC042 is a regulator of disparate phytoalexin pathways in plants.

### 3.2. NAC42-Type Transcription Factors Are Opportunistic Regulators That Coopt Lineage-Specific Genes into Pathogen-Inducible Biochemical Defenses

Phytoalexins are crucial components of plant defense, with their biosynthesis stemming from both conserved and highly specialized metabolic pathways across lineages [25,35]. We previously found that the *ANAC042* homolog, *GmNAC42-1*, encodes a protein that directly binds and activates the expression of soybean-specific phytoalexin genes [44,46,47]. This demonstrated that GmNAC42-1 is a direct regulator of phytoalexin biosynthesis in soybeans; however, the role of NAC42-type TFs in other plant species has remained unclear. Here, we found that ANAC042 directly binds and regulates genes for the synthesis of 4OH-ICN and camalexin, which are specific to Arabidopsis and a few *Brassicaceae* species, respectively. This suggests that NAC42-type proteins are opportunistic TFs that coopt biosynthetic genes from species-specific biosynthetic pathways—e.g., from the indole alkaloid pathway in *Brassicaceae* species and the isoflavonoid pathway in soybean, respectively. This could represent an adaptive mechanism where *ANAC042* and its homolog *GmNAC42-1* opportunistically coopt lineage-specific defenses in response to selective pressures, such as specialist pathogens [22,44,47]. These findings provide an exception to the paradigm that TFs have conserved roles in regulating specific specialized metabolic pathways across plant lineages [48]. They suggest that NAC42-type TFs have a more dynamic and flexible regulatory architecture. Their expression remains pathogen-inducible among soybean and Arabidopsis, yet their gene targets, at least in part, have diversified. This dynamic and flexible regulatory architecture may not be limited to NAC42-type TFs but rather shared by an as-of-yet unknown network of conserved transcription factors. Whether NAC42-type TFs evolved to first regulate conserved phytoalexin pathways, such as pathogen-inducible lignin biosynthesis and scopoletin, then coopted lineage-specific biochemical pathways remains an important topic for future investigation.

### 3.3. ANAC042 as a Member of a Cooperative Network That Regulates Phytoalexin Biosynthesis

Our results add to the growing number of studies that suggest that phytoalexin biosynthesis is regulated by a cooperative network of TFs. We found that the ANAC042 protein binds and activates the same promoters of camalexin and 4OH-ICN genes as WRKY33, ERF1, and ERF72 [14,18,19,45]. Further, same as MYB15, ANAC042 activates the PAL promoter for pathogen-inducible lignin and scopoletin biosynthesis [28] (Figure 4). All of these TFs are co-expressed in response to PAMPs such as Flg22; thus, it is tempting to speculate that all may physically interact to regulate phytoalexin biosynthetic genes.

ANAC042 also positively regulates the expression of *MYB15*. This multilayered regulation of camalexin TF and biosynthetic genes was also observed for ERF1 and ERF72, which activate the expression of *WRKY33* and *CYP71B15/PAD3* [14,18]. Recently, ERF1 and ERF72 proteins were found to physically interact with WRKY33, putatively forming transcriptional complexes, to synergistically activate camalexin gene promoters [14,18]. This has been proposed to provide a point of convergence between ethylene/JA and MAPK signaling pathways for the activation of camalexin biosynthesis [14]. The promoter of *ANAC042* is responsive to ROS, Ca^2+^ ion, kinase, and methyl jasmonate signaling [22,40]. Further, the activity of ANAC042-type TFs is inhibited by interacting JAZ1 proteins, whose transcription is upregulated in response to drought and ABA signaling [47]. Thus, ANAC042 potentially serves as a point of integration of pathogen- and abiotic stress-responsive signaling pathways that activate or suppress the expression of phytoalexin genes. Our results show that ANAC042 activates the expression of the same Trp and Phe phytoalexin gene promoters as WRKY33, ERF1, ERF72, and MYB15, respectively. This opens the possibility that all of these TFs cooperate to broadly regulate phytoalexin biosynthesis in Arabidopsis, a question that must be addressed in future research.

### 3.4. Limitations and Future Directions

Despite our clarification of ANAC042′s role in regulating phytoalexin biosynthesis in Arabidopsis, some limitations of this study warrant discussion. First, while we demonstrated the regulatory role of ANAC042 in Arabidopsis, the extent to which this regulation is conserved across other plant species remains to be elucidated. Our finding that the soybean homolog of *ANAC042* directly regulates glyceollin biosynthesis, which is a pathway that is unique to soybeans, raises the possibility that *ANAC042* broadly regulates diverse phytoalexin pathways across plant lineages. Comparative genomic and functional studies in other plant species could reveal how *ANAC042* and other phytoalexin TFs, such as *MYB15*, have evolved to regulate lineage-specific pathways. If the role of ANAC042 and its putative regulatory network is conserved, engineering crops with enhanced expression or activity of *ANAC042* and its homologs could boost phytoalexin production for pharmaceutical applications and improve pathogen resistance.

Regarding pathogen resistance, Saga et al. (2012) reported that the *anac042* mutant has reduced resistance to *A. brassicicola* and diminished camalexin accumulation [22]. Given camalexin’s role in defense against *P. syringae* and *B. cinerea*, it is plausible that *anac042* may also be susceptible to these pathogens. While our study establishes a role for ANAC042 in regulating both conserved and lineage-specific phytoalexins, future work should investigate its contribution to resistance across a broader spectrum of pathogens and further dissect the specificity of individual phytoalexins in plant immunity.

The functional interplay between ANAC042, MYB15, WRKY33, ERF1, and ERF72 requires further investigation to establish whether these TFs act independently, sequentially, and/or synergistically. Detailed analyses of how ANAC042 interacts with MYB15, WRKY33, ERF1, and ERF72 could provide insights into the hierarchical or cooperative nature of phytoalexin gene regulation. Further, the pathways that activate ANAC042, MYB15, WRKY33, ERF1, and ERF72 should be dissected to understand how upstream signaling cascades coordinate their activity in response to pathogen attack.

Finally, our study focused primarily on transcriptional regulation; future studies should explore the post-transcriptional and post-translational modifications that might influence ANAC042 activity. Other phytoalexin TFs, namely WRKY33 and ERF1, require phosphorylation by MPK3/MPK6 to achieve full DNA-binding and transactivation activities [14,19]. The ANAC042 promoter has reduced transactivation by Flg22 in the presence of a kinase inhibitor [22], suggesting that protein phosphorylation is needed for its full activation. However, it remains unknown whether phosphorylation of ANAC042 plays an important role in its activation.

## 4. Materials and Methods

### 4.1. Chemicals

Stocks (50 mg/mL) of the antibiotics, including tetracycline, kanamycin, timentin, hygromycin-B, and ampicillin (Gold Biotechnology, Olivette, MO, USA), were prepared in MilliQ-purified water. Stocks (10 mM) of camalexin (Sigma-Aldrich, St. Louis, MO, USA) and scopoletin (Cayman-Chemical, Ann Arbor, MI, USA) standards were prepared in EtOH (96%). The elicitor flg22 (5 mM; QRLSTGSRINSAKDDAAGLQIA; PhytoTech Lab, Lenexa, KS, USA) was prepared in dimethyl sulfoxide (DMSO). Phloroglucinol (1% *w*/*v*; Thermo-Fisher, Waltham, MA, USA) was prepared in 1:1 HCl:H_2_O. Other reactants and solvents were purchased from Sigma-Aldrich (St. Louis, MO, USA).

### 4.2. Cloning and Plasmid Constructs

*ANAC042-pENTR D-TOPO*, *MYB15-pENTR D-TOPO*, and *WRKY33-pENTR D-TOPO* were purchased from the Arabidopsis Biological Resource Center (ABRC). After sequencing the CDSs, entry vectors were LR recombined using LR clonase (Invitrogen, Burlington, ON, Canada) into different destination vectors, including (1) *pDEST-GADT7* for Y1H, (2) *p62GW* for luciferase transactivation assay, (3) *pGWB6* for subcellular localization, and (4) *pGWB2* for overexpression in plants.

Promoters were cloned from genomic DNA and inserted into the *pGreenII0800-LUC* vector for luciferase transactivation assay or the entry vector *pGG* for Y1H using HindIII restriction enzyme (New England Biolabs, Ipswich, MA, USA). After sequencing, entry vectors were LR recombined into *pMW#2* for Y1H.

### 4.3. Plant Materials

Arabidopsis transgenic lines were generated according to the protocol described by Zhang et al. [49]. Arabidopsis seeds were stratified for 3 days at 4 °C in darkness, followed by spreading in wet soil. Flowering plants were dipped for 10 s in a solution containing *Agrobacterium tumefaciens* strain GV3101 transformed with either *p35S::ANAC042* or *p35S::GFP::ANAC042* constructs and grown until they generated seeds. Transgenic seeds were selected on solid Murashige and Skoog (MS) medium containing kanamycin (50 mg/L), hygromycin (50 mg/L), sucrose (1% *w*/*v*), and Gelzan (2.5 g/L) at pH 5.8 for further experiments.

Arabidopsis seeds were sterilized following the protocol described by Denoux et al. [50]. The sterilized seeds were plated on a solid MS medium containing kanamycin (50 mg/L), hygromycin (50 mg/L), sucrose (1% *w*/*v*), and Gelzan (2.5 g/L) at pH 5.8. Seeds were kept in darkness at 4 °C for 3 days, exposed to cool white T5 fluorescent lights (100 μEm^2^/s) for 5–6 h, and returned to darkness at 22 °C for 3 days. Seedlings were transferred into a 16 h photoperiod using cool white T5 fluorescent lights (100 μEm^2^/s), grown for five days, and then transferred into MS liquid medium (12-well plate; 1 mL). For metabolites and gene expression measurements, seedlings were grown for four more days. A day before treatment (nine-day-old seedlings), the medium was replaced with a fresh MS medium. Ten-day-seedlings were treated with 5 μM flg22 for 12 h. The liquid medium was used for metabolite measurements and the seedlings were used for gene expression measurement. For lignin staining, seedlings were transferred into an MS liquid medium containing 5 μM flg22 (12-well plate; 1 mL) and then grown for two more days. The liquid medium was removed, and the seedlings were used for lignin staining.

*Nicotiana benthamiana* seeds were sown in a 1:3 ratio of nutrient-holding mix to soil (Berger, QC, Canada). Plants were kept under plastic domes for two weeks, after which seedlings were transplanted to individual pots and maintained under plastic domes for three days. Plants were maintained in a growth chamber with a 16 h light/8 h dark photoperiod with respective temperatures of 24 °C and 20 °C and a fan speed of 65% in a BigFoot^TM^ Series growth chamber (BioChambers, Winnipeg, MB, Canada). After the removal of the domes, plants were watered every two to three days. Four-to-five-week-old *N. benthamiana* plants were used for luciferase transactivation assays.

### 4.4. Metabolites Analyses

Metabolite analysis was conducted using a modified version of the method recently published by Parasecolo et al. [51], adapted for relative quantification purposes. Briefly, the liquid medium was extracted with ethyl acetate (1:0.5 *v*/*v* ratio) twice. The organic phase was separated and dried under nitrogen gas. The dry extract was dissolved using MeOH:H_2_O:AcOH (80:19:1 *v*/*v*/*v*) to make a solution of 20 μL/mg of dry plant material. A 20 µL aliquot of this solution was mixed with 20 µL of acetone saturated with 2,3-diaminonaphthalene (DAN) and containing daidzein at a concentration of 0.62 µM, which was used as an internal standard to normalize the ion intensities of the metabolites of interest. A 4 µL aliquot of the resulting mixture was applied to a Teflon-coated slide (Tekdon Incorporated, Myakka City, FL, USA), allowed to dry, and subsequently analyzed. MALDI-HRMS experiments were conducted using a Q-Exactive mass spectrometer (Thermo Fisher Scientific) equipped with a Spectroglyph ESI/MALDI ion source (Spectroglyph LLC, Kennewick, WA, USA). The acquisitions were performed with Tune acquisition software version 2.9 build 2926 operated with a resolving power of 70,000 at *m*/*z* 200, a maximum injection time of 200 ms, and an *m*/*z* range of 100–1000, and AGC target = 1 × 10^6^. The laser current was set to 1.8 A, with a repetition rate of 300 Hz. Each sample was analyzed in triplicate, with each analysis performed by scanning the sample for 30 s. Metabolites were identified based on a mass error of less than 5 ppm. Blank analyses were conducted by acquiring triplicate measurements of the blank matrix, both with and without the internal standard, to exclude the possibility that the identified *m*/*z* values were analytical artifacts or interferences affecting the intensities of the *m*/*z* of interest. The acquired ion intensities were extracted using Thermo Xcalibur Qual Browser version 4.1.50.

### 4.5. Lignin Staining

Lignin staining was performed according to the protocol of Chezem et al. [28]. Briefly, 12-day-old seedlings (*n* = 10–15) treated with either 5 μM Flg22 or dimethyl sulfoxide (DMSO) for 48 h were vacuum infiltrated with the fixative solution 3:1 95% EtOH: AcOH for five min. The solution was replaced with a new 3:1 95% EtOH: AcOH and placed on an orbital-shaking platform for 1 h at 80 rpm. This process was repeated twice. The fixative solution was then replaced sequentially with 75% ethanol (orbital-shaking at 80 rpm; 30 min), 50% ethanol (orbital-shaking at 80 rpm; 30 min), and sterile ddH₂O (orbital-shaking at 80 rpm; overnight). The following day, seedlings were stained in 1% phloroglucinol dissolved in 50% (*v*/*v*) HCl for five minutes and photographed under a Wild M3B stereo microscope (Leica, Wetzlar, HE, Germany).

### 4.6. RNA Extraction and Gene Expression Measurements (qRT-PCR)

Seedlings were snap-frozen in liquid nitrogen, lyophilized, weighed (for metabolites analysis), and homogenized in a Retsch Mixer Mill MM 400 (Verder Scientific, ON, Canada) at 30/s frequency for 2 min. Total RNA was isolated using HiPure Total RNA Mini Kit (GeneBio System, Burlington, ON, Canada) following the manufacturer’s protocol. Complementary DNA was obtained using a DNA synthesis kit (GeneBio System, Burlington, ON, Canada) following the manufacturer’s protocol. qRT-PCR was conducted on a Bio-Rad CFX96 machine (Bio-Rad Laboratories, Mississauga, ON, Canada) using GB-AmpTM Sybr Green qPCR mix (GeneBio System, Burlington, ON, Canada). The thermal cycling was as follows: initial denaturation at 95 °C for 3 min, followed by 40 cycles of 95 °C for 5 s and 60 °C for 30 s, and a melt curve analysis was included from 65 °C to 95 °C. UBIQUITIN2 served as the internal reference for the transcripts. The comparative CT method: expression = 2^−[Ct(gene)−Ct(UBIQUITIN2)]^ was used to analyze qPCR data. Primers used in this study are listed in Supplemental Table S4.

### 4.7. Subcellular Localization

Seedlings (ten-day-old) of *p35S::GFP::ANAC042*-18-12 or *anac042-1* (negative control) were mounted on the 10% glycerol and stained by 4′,6-diamidino-2-phenylindole (DAPI; 6 μg/mL) (Cayman Chemical, Ann Arbor, MI, USA). Six seedlings per genotype were analyzed. Confocal laser microscopy (LMS 700, Carl Zeiss, Oberkochen, Germany) was used to observe the GFP fluorescence and Zen Black v2.3 SP1 software was used to modify the image. Excitation and emission spectra were 488 nm and 500–550 nm for GFP and 405 nm and 358–461 nm for DAPI, respectively.

### 4.8. Y1H

Yeast strain YM4271 (Saccharomyces cerevisiae; MATa, ura3–52, his3–200, lys2–801, ade2–101, ade5, trp1–901, leu2–3, 112, tyr1–501, gal4D, gal80D, ade5::hisG) was transformed with the linear *pMW#2* (*pPAL1::HIS3*, *pCYP71A12::HIS3*, and *pCYP71B15::HIS3*), and selected in SD media lacking histidine. Then, yeast bait strains were transformed with one of the recombinant plasmids *pDEST-GADT7-TF* (*pAD-ANAC0422*, *pAD-WRKY33*, or *pAD-MYB15*) and selected in media lacking leucine. For the *HIS3* reporter gene, positive PDIs were assessed by differential growth in media containing 3-amino-1,2,4-triazol (3AT) and lacking histidine and leucine.

### 4.9. Luciferase Transactivation Assay

Promoters (*pPAL1::LUC*, *pCYP71A12::LUC*, and *pCYP71B15::LUC*) and TFs (*p35S::ANAC042*, *p35S::WRKY33*, and *p35S::MYB15*) were transformed into chemically competent *A. tumefaciens* strain EHA105 (*pSoup-p19*) (GoldBio, St. Louis, MO, USA), selected on Luria-Bertani (LB) medium with kanamycin and tetracycline, and verified by colony PCR. Agrobacteria were freshly streaked and incubated at 30 °C for 16 h, then resuspended in buffer (100 mM MgCl_2_, 100 mM MES pH 5.7, 100 µM acetosyringone) to an OD_600_ of 0.8. Samples harboring promoter and transcription factor constructs were combined in a 1:1 ratio and then infiltrated into *N. benthamiana* plants. Infiltrated plants were kept in the darkness for 16 h, followed by regular growth conditions (16 h light/8 h dark). At two days post-infiltration, 15 to 20 mg of plant tissue was ground at homogenized in an MM400 mixer mill (Retsch, Newtown, CT, USA) at 30/s frequency for one min and resuspended in a 10x volume of Passive Lysis Buffer (Promega, Madison, WI, USA). Firefly and Renilla luciferase activity of the lysate was measured using a Synergy H4 Hybrid Multi-Mode Microplate Reader (BioTek, Winooski, VT, USA) equipped with the Gen5 software (Version 2.00.17) and the Dual-Luciferase Reporter Assay System (Promega, Madison, WI, USA).

### 4.10. Statistical Analysis

The Tukey post hoc test in one-way ANOVA was used to analyze whether any statistically significant differences existed between group means at α = 0.05. All trials were independently repeated at least three times.

### 4.11. Accession Numbers

ANAC042, AT2G43000; WRKY33, AT2G38470; MYB15, AT3G23250; ERF1, AT3G23240; ERF72, AT3G16770; EMB1144, AT1G48850; PAL1, AT2G37040; F6′H, AT3G13610; CAD5, AT4G34230; COMT, AT5G54160; F5H, AT4G36220; CYP79B2, AT4G39950; CYP71A12, AT2G30750; FOX1, AT1G26380; CYP82C2, AT4G3197; CYP71A13, AT2G30770; CYP71B15, AT3G26830.

## 5. Conclusions

Our findings establish ANAC042 as a central regulator of phytoalexin biosynthesis in Arabidopsis, bridging distinct metabolic pathways and expanding the paradigm of TF roles in specialized metabolism. By uncovering its interactions with *MYB15*, *ERF1*, and *ERF72*, we lay the groundwork for future explorations into the evolution, function, and application of this versatile regulatory network.

The findings from this study raise intriguing questions about the flexibility and adaptability of TF networks. We postulate that ANAC042 serves as a molecular integrator capable of coopting conserved and lineage-specific genes into a cohesive defense response. This opportunistic behavior might reflect an evolutionary strategy to enhance fitness under diverse environmental pressures. Testing this hypothesis will require functional studies of ANAC042 homologs in a range of plant species and environmental contexts.

## Data Availability

Data is contained within the article and supplementary material.

## Supplementary Materials

The supporting information can be downloaded at https://www.mdpi.com/article/10.3390/ijms26083683/s1.
