# Supplementary material for: ANAC042 Regulates the Biosynthesis of Conserved- and Lineage-Specific Phytoalexins in Arabidopsis
Source: Int J Mol Sci. 2025 Apr 13;26(8):3683. doi: 10.3390/ijms26083683 (PMC12027767; doi:10.3390/ijms26083683)
Supplement: Supplementary file 1 [file ijms-26-03683-s001.zip › Table S1. Statistical results for ANAC042 gene expression and metabolite measurements shown in Figure 1A_1B_1C..pdf]

Camalexin

Anova: Single Factor

SUMMARY

Groups	Count	Sum	Average	Variance	SE
WT	6	0.05553422	0.009255703	9.2929E-07	0.00039355
anac042-1	6	0.023774	0.003962333	1.7605E-06	0.00054168
35S::ANAC042 anac042-1 2-23	6	0.09818	0.016363333	3.7822E-06	0.00079395
35S::ANAC042 anac042-1 2-26	6	0.12711	0.021185	2.6539E-06	0.00066507

ANOVA

Source of Variation	SS	df	MS	F	P-value	F crit
Between Groups	0.00104175	3	0.00034725	152.205538	6.14299E-14	3.09839121
Within Groups	4.56291E-05	20	2.28145E-06			
Total	0.001087379	23				

vs	WT	anac042-1	35S::ANAC042 anac042-1 2-23
anac042-1	8.58424367		
35S::ANAC042 anac042-1 2-23	11.52642416	20.11066783	
35S::ANAC042 anac042-1 2-26	19.34570782	27.92995149	7.819283662

Critical Value = 3.91

4OH-ICN

Anova: Single Factor

SUMMARY

Groups	Count	Sum	Average	Variance	SE
WT	6	0.528658	0.088109667	6.3492E-05	0.003253
anac042-1	6	0.288043	0.048007167	4.746E-05	0.00281247
35S::ANAC042 anac042-1 2-23	6	0.47931519	0.079885865	7.4451E-05	0.00352256
35S::ANAC042 anac042-1 2-26	6	0.52875797	0.088126328	2.1029E-05	0.00187213

ANOVA

Source of Variation	SS	df	MS	F	P-value	F crit
Between Groups	0.006554318	3	0.002184773	42.3340284	7.5267E-09	3.09839121
Within Groups	0.001032159	20	5.1608E-05			
Total	0.007586477	23				

vs	WT	anac042-1	35S::ANAC042 anac042-1 2-23
anac042-1	13.67378469		
35S::ANAC042 anac042-1 2-23	2.804076886	10.8697078	
35S::ANAC042 anac042-1 2-26	0.005681143	13.67946583	2.809758029

Critical Value = 3.91

Scopoletin

Anova: Single Factor

SUMMARY

Groups	Count	Sum	Average	Variance	SE
WT	6	0.04557	0.007595	6.5647E-07	0.00033077
anac042-1	6	0.02137	0.003561667	3.6402E-07	0.00024631
35S::ANAC042 anac042-1 2-23	6	0.03456	0.00576	6.1552E-07	0.00032029
35S::ANAC042 anac042-1 2-26	6	0.03730448	0.006217413	1.5536E-06	0.00050885

ANOVA

Source of Variation	SS	df	MS	F	P-value	F crit
Between Groups	5.04415E-05	3	1.68138E-05	21.0860615	2.09272E-06	3.09839121
Within Groups	1.59478E-05	20	7.9739E-07			
Total	6.63893E-05	23				

vs	WT	anac042-1	35S::ANAC042 anac042-1 2-23
anac042-1	11.06379992		
35S::ANAC042 anac042-1 2-23	5.033571782	6.030228138	
35S::ANAC042 anac042-1 2-26	3.778845434	7.284954485	1.254726347

Critical Value = 3.91

Monolignol H

Anova: Single Factor

SUMMARY

Groups	Count	Sum	Average	Variance	SE
WT	6	0.02474265	0.004123775	4.1253E-07	0.0002622
anac042-1	6	0.01479067	0.002465112	1.4596E-07	0.000156
35S::ANAC042 anac042-1 2-23	6	0.02678774	0.004464623	2.6626E-07	0.0002107
35S::ANAC042 anac042-1 2-26	6	0.0241777	0.004029617	2.0509E-07	0.0001849

ANOVA

Source of Variation	SS	df	MS	F	P-value	F crit
Between Groups	1.42667E-05	3	4.75558E-06	18.47107	5.54744E-06	3.09839121
Within Groups	5.14922E-06	20	2.57461E-07			
Total	1.9416E-05	23				

vs	WT	anac042-1	35S::ANAC042 anac042-1 2-23
anac042-1	8.007152994		
35S::ANAC042 anac042-1 2-23	1.645436237	9.652589231	

35S::ANAC042 anac042-1 2-26	0.454546842	7.552606152	2.099983079

Critical Value = 3.91

Monolignol G

Anova: Single Factor

SUMMARY

Groups	Count	Sum	Average	Variance	SE
WT	6	0.00865254	0.00144209	2.7373E-08	0.0000675
anac042-1	6	0.00509971	0.000849952	1.7058E-08	0.0000533
35S::ANAC042 anac042-1 2-23	6	0.00965807	0.001609678	1.1752E-07	0.0001399
35S::ANAC042 anac042-1 2-26	6	0.00898283	0.001497138	9.2618E-08	0.0001242

ANOVA

Source of Variation	SS	df	MS	F	P-value	F crit
Between Groups	2.08567E-06	3	6.95222E-07	10.9240975	0.000183068	3.09839121
Within Groups	1.27282E-06	20	6.36412E-08			
Total	3.35849E-06	23				

vs	WT	anac042-1	35S::ANAC042 anac042-1 2-23
anac042-1	5.749495878		
35S::ANAC042 anac042-1 2-23	1.627235356	7.376731234	
35S::ANAC042 anac042-1 2-26	0.53450376	6.283999638	1.092731596

Critical Value = 3.91

Monolignol S

Anova: Single Factor

SUMMARY

Groups	Count	Sum	Average	Variance	SE
WT	6	0.0006131	0.000102183	8.1833E-10	0.0000117
anac042-1	6	0.000302	5.03333E-05	3.9364E-10	0.0000081
35S::ANAC042 anac042-1 2-23	6	0.0001677	0.00002795	1.0744E-09	0.0000134
35S::ANAC042 anac042-1 2-26	6	0.0001484	2.47333E-05	8.5249E-10	0.0000119

ANOVA

Source of Variation	SS	df	MS	F	P-value	F crit
Between Groups	2.30464E-08	3	7.68212E-09	9.78978367	0.000349991	3.09839121
Within Groups	1.56942E-08	20	7.84708E-10			
Total	3.87405E-08	23				

vs	WT	anac042-1	35S::ANAC042 anac042-1 2-23
anac042-1	4.533884832		
35S::ANAC042 anac042-1 2-23	6.491135661	1.957250829	
35S::ANAC042 anac042-1 2-26	6.772408491	2.238523659	0.28127283

Critical Value = 3.91

ANAC042

Anova: Single Factor

SUMMARY

Groups	Count	Sum	Average	Variance	SE
WT	9	0.062415989	0.00693511	5.1465E-06	0.0007562
anac042-1	9	0.017129972	0.00190333	7.6346E-08	0.0000921
35S::ANAC042 anac042-1 2-23	9	0.132692535	0.014743615	7.2442E-06	0.0008971
35S::ANAC042 anac042-1 2-26	9	0.304344773	0.033816086	3.8007E-05	0.0020499

ANOVA

Source of Variation	SS	df	MS	F	P-value	F crit
Between Groups	0.005300853	3	0.001766951	140.029078	1.77157E-18	2.90111958
Within Groups	0.000403791	32	1.26185E-05			
Total	0.005704643	35				

vs	WT	anac042-1	35S::ANAC042 anac042-1 2-23
anac042-1	4.249518781		
35S::ANAC042 anac042-1 2-23	6.594563132	10.84408191	
35S::ANAC042 anac042-1 2-26	22.7019503	26.95146909	16.10738717

Critical Value = 3.83
