# Supplementary material for: ANAC042 Regulates the Biosynthesis of Conserved- and Lineage-Specific Phytoalexins in Arabidopsis
Source: Int J Mol Sci. 2025 Apr 13;26(8):3683. doi: 10.3390/ijms26083683 (PMC12027767; doi:10.3390/ijms26083683)
Supplement: Supplementary file 1 [file ijms-26-03683-s001.zip › Table S2. Statistical results for gene expression measurements shown in Figure 2..pdf]

qEMB114

Anova: Single Factor

SUMMARY

Groups	Count	Sum	Average	Variance	SE
WT	9	1.329124475	0.147680497	0.00526771	0.024193
anac042-1	9	0.368864076	0.040984897	0.00034731	0.006212
35S::ANAC042 anac042-1 2-23	9	1.860214948	0.20669055	0.00743373	0.028739
35S::ANAC042 anac042-1 2-26	9	1.520599325	0.168955481	0.00584761	0.025489

ANOVA

Source of Variation	SS	df	MS	F	P-value	F crit
Between Groups	0.136299445	3	0.045433148	9.6173327	0.000112199	2.90111958
Within Groups	0.15117089	32	0.00472409			
Total	0.287470335	35				

vs	WT	anac042-1	35S::ANAC042 anac042-1 2-23
anac042-1	4.657026057		
35S::ANAC042 anac042-1 2-23	2.575657784	7.23268384	
35S::ANAC042 anac042-1 2-26	0.928605792	5.585631849	1.647051991

Critical Value = 3.83

qPAL

Anova: Single Factor

SUMMARY

Groups	Count	Sum	Average	Variance	SE
WT	9	2.822020943	0.313557883	0.00631345	0.0264857
anac042-1	9	0.594404099	0.0660449	0.00177538	0.0140451
35S::ANAC042 anac042-1 2-23	9	13.46323033	1.495914481	0.53198431	0.2431242
35S::ANAC042 anac042-1 2-26	9	12.68411152	1.409345725	0.56559717	0.2506873

ANOVA

Source of Variation	SS	df	MS	F	P-value	F crit
Between Groups	14.66203292	3	4.887344306	17.6810184	5.99345E-07	2.90111958
Within Groups	8.845362515	32	0.276417579			
Total	23.50739543	35				

vs	WT	anac042-1	35S::ANAC042 anac042-1 2-23
anac042-1	4.504210851		
35S::ANAC042 anac042-1 2-23	6.746630187	8.158960918	
35S::ANAC042 anac042-1 2-26	6.252661288	7.664992019	0.493968899

Critical Value = 3.83

qF6'H

Anova: Single Factor

SUMMARY

Groups	Count	Sum	Average	Variance	SE
WT	9	22.9832859	2.553698433	0.88533071	0.31364005
anac042-1	9	3.479784808	0.386642756	0.06301256	0.08367434
35S::ANAC042 anac042-1 2-23	9	18.31589992	2.035099991	2.01939772	0.47368505
35S::ANAC042 anac042-1 2-26	9	22.96443896	2.551604329	2.08886471	0.48176351

ANOVA

Source of Variation	SS	df	MS	F	P-value	F crit
Between Groups	28.43173267	3	9.477244223	7.49692167	0.000617648	2.90111958
Within Groups	40.45284565	32	1.264151427			
Total	68.88457832	35				

vs	WT	anac042-1	35S::ANAC042 anac042-1 2-23
anac042-1	5.782182229		
35S::ANAC042 anac042-1 2-23	1.383734958	4.398447271	
35S::ANAC042 anac042-1 2-26	0.005587531	5.776594698	1.378147427

Critical Value = 3.83

qCAD5

Anova: Single Factor

SUMMARY

Groups	Count	Sum	Average	Variance	SE
WT	9	1.152812313	0.128090257	0.00120903	0.0115904
anac042-1	9	0.120979666	0.013442185	6.2914E-05	0.002644
35S::ANAC042 anac042-1 2-23	9	0.898843769	0.09987153	0.00083684	0.0096428
35S::ANAC042 anac042-1 2-26	9	0.548475031	0.06094167	0.00032108	0.0059729

ANOVA

Source of Variation	SS	df	MS	F	P-value	F crit
Between Groups	0.066805147	3	0.022268382	36.657764	1.81883E-10	2.90111958
Within Groups	0.019438944	32	0.000607467			
Total	0.086244091	35				

vs	WT	anac042-1	35S::ANAC042 anac042-1 2-23
anac042-1	13.95489783		
35S::ANAC042 anac042-1 2-23	3.43476734	10.52013049	
35S::ANAC042 anac042-1 2-26	8.173287646	5.781610187	4.738520306

Critical Value = 3.83

qCOMT

Anova: Single Factor

SUMMARY

Groups	Count	Sum	Average	Variance	SE
WT	9	5.967791598	0.663087955	0.18236222	0.1423463
anac042-1	9	0.393648259	0.043738695	0.00057552	0.00799667
35S::ANAC042 anac042-1 2-23	9	3.514180179	0.390464464	0.04064899	0.06720532
35S::ANAC042 anac042-1 2-26	9	1.836792289	0.204088032	0.00322956	0.01894307

ANOVA

Source of Variation	SS	df	MS	F	P-value	F crit
Between Groups	1.910845904	3	0.636948635	11.2328553	3.40952E-05	2.90111958
Within Groups	1.8145303	32	0.056704072			
Total	3.725376204	35				

vs	WT	anac042-1	35S::ANAC042 anac042-1 2-23
anac042-1	7.80278705		
35S::ANAC042 anac042-1 2-23	3.434609812	4.368177238	
35S::ANAC042 anac042-1 2-26	5.782647835	2.020139215	2.348038023

Critical Value = 3.83

qF5H

Anova: Single Factor

SUMMARY

Groups	Count	Sum	Average	Variance	SE
WT	9	11.64533503	1.293926115	0.07130178	0.08900798
anac042-1	9	2.806644195	0.311849355	0.02663432	0.05440008
35S::ANAC042 anac042-1 2-23	9	8.238583185	0.915398132	0.1323541	0.12126834
35S::ANAC042 anac042-1 2-26	9	10.87496952	1.208329946	0.05572833	0.07868949

ANOVA

Source of Variation	SS	df	MS	F	P-value	F crit
Between Groups	5.32989568	3	1.776631893	24.8463887	1.71618E-08	2.90111958
Within Groups	2.288148239	32	0.071504632			
Total	7.618043918	35				

vs	WT	anac042-1	35S::ANAC042 anac042-1 2-23
anac042-1	11.01791961		
35S::ANAC042 anac042-1 2-23	4.246705612	6.771213996	
35S::ANAC042 anac042-1 2-26	0.960303454	10.05761615	3.286402158

Critical Value = 3.83

qCYP79B2

Anova: Single Factor

SUMMARY

Groups	Count	Sum	Average	Variance	SE
WT	9	2.238842257	0.248760251	0.04094531	0.0674498
anac042-1	9	0.236774367	0.026308263	0.0008934	0.0099633
35S::ANAC042 anac042-1 2-23	9	0.95779684	0.106421871	0.00461051	0.0226336
35S::ANAC042 anac042-1 2-26	9	3.11701876	0.346335418	0.14425374	0.1266025

ANOVA

Source of Variation	SS	df	MS	F	P-value	F crit
Between Groups	0.552735212	3	0.184245071	3.86454543	0.018210477	2.90111958
Within Groups	1.52562374	32	0.047675742			
Total	2.078358952	35				

vs	WT	anac042-1	35S::ANAC042 anac042-1 2-23
anac042-1	12.28648995		
35S::ANAC042 anac042-1 2-23	7.861647311	4.424842636	
35S::ANAC042 anac042-1 2-26	1.340638935	17.67577112	13.25092848

Critical Value = 3.83

qCYP71A12

Anova: Single Factor

SUMMARY

Groups	Count	Sum	Average	Variance	SE
WT	9	0.070027579	0.007780842	1.5372E-05	0.00130688
anac042-1	9	0.011981277	0.001331253	9.8088E-07	0.00033013
35S::ANAC042 anac042-1 2-23	9	0.422757504	0.046973056	0.00190478	0.01454792
35S::ANAC042 anac042-1 2-26	9	0.740475948	0.082275105	0.00022253	0.00497252

ANOVA

Source of Variation	SS	df	MS	F	P-value	F crit
Between Groups	0.038268761	3	0.012756254	23.802719	2.74932E-08	2.90111958
Within Groups	0.017149306	32	0.000535916			
Total	0.055418067	35				

vs	WT	anac042-1	35S::ANAC042 anac042-1 2-23
anac042-1	0.835805088		
35S::ANAC042 anac042-1 2-23	5.078936276	5.914741364	
35S::ANAC042 anac042-1 2-26	9.653744406	10.48954949	4.57480813

Critical Value = 3.83

qFOX1

Anova: Single Factor

SUMMARY

Groups	Count	Sum	Average	Variance	SE
WT	9	9.790568383	1.087840931	0.56533688	0.2506296
anac042-1	9	1.604955661	0.178328407	0.0216944	0.0490967
35S::ANAC042 anac042-1 2-23	9	8.516170559	0.946241173	0.11033732	0.1107235
35S::ANAC042 anac042-1 2-26	9	8.233604081	0.914844898	0.33204822	0.1920788

ANOVA

Source of Variation	SS	df	MS	F	P-value	F crit
Between Groups	4.523227732	3	1.507742577	5.85862817	0.00262453	2.90111958
Within Groups	8.235334463	32	0.257354202			
Total	12.75856219	35				

vs	WT	anac042-1	35S::ANAC042 anac042-1 2-23
anac042-1	5.378538813		
35S::ANAC042 anac042-1 2-23	0.837371421	4.541167392	
35S::ANAC042 anac042-1 2-26	1.023038007	4.355500806	0.185666586

Critical Value = 3.83

qCYP82C2

Anova: Single Factor

SUMMARY

Groups	Count	Sum	Average	Variance	SE
WT	9	4.787977445	0.531997494	0.10357439	0.10727658
anac042-1	9	1.34861828	0.149846476	0.00923205	0.03202785
35S::ANAC042 anac042-1 2-23	9	4.480529972	0.497836664	0.12564767	0.11815605
35S::ANAC042 anac042-1 2-26	9	7.321329981	0.813481109	0.43547032	0.21996725

ANOVA

Source of Variation	SS	df	MS	F	P-value	F crit
Between Groups	1.997052531	3	0.665684177	3.95109102	0.016670339	2.90111958
Within Groups	5.391395332	32	0.168481104			
Total	7.388447862	35				

vs	WT	anac042-1	35S::ANAC042 anac042-1 2-23
anac042-1	5.250142479		
35S::ANAC042 anac042-1 2-23	0.469315055	4.780827424	
35S::ANAC042 anac042-1 2-26	3.86713371	9.11727619	4.336448766

Critical Value = 3.83

qCYP71A13

Anova: Single Factor

SUMMARY

Groups	Count	Sum	Average	Variance	SE
WT	9	0.1642165	0.018246278	3.1622E-05	0.00187445
anac042-1	9	0.070718686	0.007857632	4.4954E-06	0.00070675
35S::ANAC042 anac042-1 2-23	9	0.171668845	0.019074316	3.3473E-05	0.00192852
35S::ANAC042 anac042-1 2-26	9	0.272157584	0.030239732	0.00015022	0.00408547

ANOVA

Source of Variation	SS	df	MS	F	P-value	F crit
Between Groups	0.002258756	3	0.000752919	13.7012876	6.43348E-06	2.90111958
Within Groups	0.001758477	32	5.49524E-05			
Total	0.004017232	35				

vs	WT	anac042-1	35S::ANAC042 anac042-1 2-23
anac042-1	4.20423403		
35S::ANAC042 anac042-1 2-23	0.335103049	4.539337079	
35S::ANAC042 anac042-1 2-26	4.853691904	9.057925934	4.518588855

Critical Value = 3.83

qCYP71B15

Anova: Single Factor

SUMMARY

Groups	Count	Sum	Average	Variance	SE
WT	9	13.96737194	1.551930216	0.72972294	0.28474607
anac042-1	9	3.176495262	0.352943918	0.07725511	0.09264934
35S::ANAC042 anac042-1 2-23	9	33.29044001	3.698937778	1.5428045	0.41403227
35S::ANAC042 anac042-1 2-26	9	58.10279128	6.455865697	8.79276136	0.98841969

ANOVA

Source of Variation	SS	df	MS	F	P-value	F crit
Between Groups	193.8099905	3	64.60333015	23.191591	3.64678E-08	2.90111958
Within Groups	89.14035127	32	2.785635977			
Total	282.9503417	35				

vs	WT	anac042-1	35S::ANAC042 anac042-1 2-23
anac042-1	2.15512911		
35S::ANAC042 anac042-1 2-23	4.807604599	6.014287891	
35S::ANAC042 anac042-1 2-26	8.814624598	10.96975371	4.955465817

Critical Value = 3.83

qWRKY33

Anova: Single Factor

SUMMARY

Groups	Count	Sum	Average	Variance	SE
WT	9	1.234842962	0.137204774	0.00382294	0.02061
anac042-1	9	1.258175409	0.139797268	0.00322127	0.0189187
35S::ANAC042 anac042-1 2-23	9	0.960506218	0.106722913	0.00082632	0.0095819
35S::ANAC042 anac042-1 2-26	9	1.45280317	0.161422574	0.00089171	0.0099538

ANOVA

Source of Variation	SS	df	MS	F	P-value	F crit
Between Groups	0.01367097	3	0.00455699	2.08028713	0.122391528	2.90111958
Within Groups	0.07009786	32	0.002190558			
Total	0.08376883	35				

vs	WT	anac042-1	35S::ANAC042 anac042-1 2-23
anac042-1	0.16617345		
35S::ANAC042 anac042-1 2-23	1.953823532	2.119996983	
35S::ANAC042 anac042-1 2-26	1.5523104	1.38613695	3.506133932

Critical Value = 3.83

qMYB15

Anova: Single Factor

SUMMARY

Groups	Count	Sum	Average	Variance	SE
WT	9	0.057640279	0.006404475	7.5697E-07	0.00029001
anac042-1	9	0.018389344	0.00204326	2.663E-07	0.00017202
35S::ANAC042 anac042-1 2-23	9	0.03805561	0.004228401	5.7942E-06	0.00080237
35S::ANAC042 anac042-1 2-26	9	0.045927459	0.005103051	8.9909E-07	0.00031607

ANOVA

Source of Variation	SS	df	MS	F	P-value	F crit
Between Groups	9.07906E-05	3	3.02635E-05	15.6875501	1.88427E-06	2.90111958
Within Groups	6.17326E-05	32	1.92914E-06			
Total	0.000152523	35				

vs	WT	anac042-1	35S::ANAC042 anac042-1 2-23
anac042-1	9.419905206		
35S::ANAC042 anac042-1 2-23	4.70016132	4.719743886	
35S::ANAC042 anac042-1 2-26	2.810981615	6.608923591	1.889179705

Critical Value = 3.83

qERF1

Anova: Single Factor

SUMMARY

Groups	Count	Sum	Average	Variance	SE
WT	9	0.148008209	0.016445357	1.9379E-05	0.00146737
anac042-1	9	0.064470953	0.007163439	4.3244E-06	0.00069318
35S::ANAC042 anac042-1 2-23	9	0.099948564	0.011105396	7.4213E-06	0.00090807
35S::ANAC042 anac042-1 2-26	9	0.123858532	0.013762059	4.5555E-05	0.00224983

ANOVA

Source of Variation	SS	df	MS	F	P-value	F crit
Between Groups	0.000423018	3	0.000141006	7.35557585	0.000696469	2.90111958
Within Groups	0.000613438	32	1.91699E-05			
Total	0.001036456	35				

vs	WT	anac042-1	35S::ANAC042 anac042-1 2-23
anac042-1	6.359875097		
35S::ANAC042 anac042-1 2-23	3.658886534	2.700988563	
35S::ANAC042 anac042-1 2-26	1.838568072	4.521307025	1.820318462

Critical Value = 3.83

qERF72

Anova: Single Factor

SUMMARY

Groups	Count	Sum	Average	Variance	SE
WT	9	1.889049186	0.209894354	0.01450735	0.04014882
anac042-1	9	0.704050466	0.07822783	0.00217761	0.01555495
35S::ANAC042 anac042-1 2-23	9	1.936559347	0.215173261	0.00257531	0.01691584
35S::ANAC042 anac042-1 2-26	9	1.445118699	0.160568744	0.00157912	0.01324603

ANOVA

Source of Variation	SS	df	MS	F	P-value	F crit
Between Groups	0.108703545	3	0.036234515	6.9550067	0.000983469	2.90111958
Within Groups	0.166715078	32	0.005209846			
Total	0.275418623	35				

vs	WT	anac042-1	35S::ANAC042 anac042-1 2-23
anac042-1	5.472479905		
35S::ANAC042 anac042-1 2-23	0.219408174	5.691888078	
35S::ANAC042 anac042-1 2-26	2.050129358	3.422350546	2.269537532

Critical Value = 3.83
