# Supplementary material for: ANAC042 Regulates the Biosynthesis of Conserved- and Lineage-Specific Phytoalexins in Arabidopsis
Source: Int J Mol Sci. 2025 Apr 13;26(8):3683. doi: 10.3390/ijms26083683 (PMC12027767; doi:10.3390/ijms26083683)
Supplement: Supplementary file 1 [file ijms-26-03683-s001.zip › Table S3. Statistical results for luciferase activity measurements shown in Figure 3C..pdf]

pCYP71B15

Anova: Single Factor

SUMMARY

Groups	Count	Sum	Average	Variance	SE
Vector	15	40.78614881	2.719076587	3.14272514	0.4577281
p35S::WRKY33	15	1067.001556	71.13343704	580.399804	6.22039551
p35S::ANAC042	15	562.816668	37.5211112	948.011781	7.94989216

ANOVA

Source of Variation	SS	df	MS	F	P-value	F crit
Between Groups	35107.47389	2	17553.73695	34.3841615	1.43057E-09	3.21994229
Within Groups	21441.76035	42	510.5181037			
Total	56549.23425	44				

vs	Vector	*p35S::WRKY33*
p35S::WRKY33	11.72701086	
p35S::ANAC042	5.965470336	3.210982478

Critical Value = 3.43

pCYP71A12

Anova: Single Factor

SUMMARY

Groups	Count	Sum	Average	Variance	SE
Vector	15	16.09951297	1.073300864	0.34770405	0.15225068
p35S::WRKY33	15	32.71775526	2.181183684	0.52724349	0.18748218
p35S::ANAC042	15	38.97256149	2.598170766	0.50061443	0.18268633

ANOVA

Source of Variation	SS	df	MS	F	P-value	F crit
Between Groups	18.63255392	2	9.316276962	20.3181183	6.72441E-07	3.21994229
Within Groups	19.25786763	42	0.458520658			
Total	37.89042156	44				

vs	Vector	*p35S::WRKY33*
p35S::WRKY33	6.336654141	
p35S::ANAC042	8.721656303	2.385002163

Critical Value = 3.43

pPAL1

Anova: Single Factor

SUMMARY

Groups	Count	Sum	Average	Variance	SE
Vector	15	2.376104065	0.158406938	0.0130315	0.02947485
p35S::MYB15	15	11.76050206	0.784033471	0.24328463	0.12735374
p35S::ANAC042	15	10.63677367	0.709118244	0.07296445	0.06974451

ANOVA

Source of Variation	SS	df	MS	F	P-value	F crit
Between Groups	3.501518968	2	1.750759484	15.9507689	7.02283E-06	3.21994229
Within Groups	4.609928135	42	0.109760194			
Total	8.111447103	44				

vs	Vector	*p35S::WRKY33*
p35S::WRKY33	7.313720447	
p35S::ANAC042	6.437943936	0.875776511

Critical Value = 3.43
