# Supplementary material for: ANAC042 Regulates the Biosynthesis of Conserved- and Lineage-Specific Phytoalexins in Arabidopsis
Source: Int J Mol Sci. 2025 Apr 13;26(8):3683. doi: 10.3390/ijms26083683 (PMC12027767; doi:10.3390/ijms26083683)
Supplement: Supplementary file 1 [file ijms-26-03683-s001.zip › Table S4. List of Oligonucleotides used in this study.pdf]

Table S4. List of Oligonucleotides used in this study

Oligo name	Accession Number	Sequence (5' to 3')	Usage
cANAC042f	AT2G43000	CTTCCTGGGTTCAGGTTTCA	PCR
cANAC042r	AT2G43000	CGGAATCTAAAGCTGCTGGT	PCR
qANAC042f	AT2G43000	TCTTGGTTCAGCCGGTAAAG	qRT-PCR
qANAC042r	AT2G43000	TGAGCTGGAGAGTCGGTTTT	qRT-PCR
cWRKY33f	AT2G38470	GGAAGCCACAACCATCCTAA	PCR
cWRKY33r	AT2G38470	TGTCGTGTGATGCTCTCTCC	PCR
qWRKY33f	AT2G38470	TGGAGAGAGCATCACACGAC	qRT-PCR
qWRKY33r	AT2G38470	GTGCTCTGTTTGTGGCGTAA	qRT-PCR
cMYB15f	AT3G23250	TAAACGTGGCAATTTCACCA	PCR
cMYB15r	AT3G23250	CGATATCCGCACCAAAAGTT	PCR
qMYB15f	AT3G23250	CTTGGCAATAGATGGTCAGC	qRT-PCR
qMYB15r	AT3G23250	TGGTTGATAATCTTCGAGTC	qRT-PCR
qERF1f	AT3G23240	GGATGGTTGTTCTCCGGTTG	qRT-PCR
qERF1r	AT3G23240	ATCTAACTTCACGGAGCGGT	qRT-PCR
qERF72f	AT3G16770	TCCACCTCCAAACTCCATCC	qRT-PCR
qERF72r	AT3G16770	TTCCTCTTCCTCCGTTTCCC	qRT-PCR
qACTIN2f	AT3G18780	GCAGAGCGGGAAATTGTAAG	qRT-PCR
qACTIN2r	AT3G18780	TTCTCGATGGAAGAGCTGGT	qRT-PCR
pPAL1f-HindIII	AT2G37040	GGATAAGCTTAACTTGAGTGTTTCGTGTGTT	Cloning / Y1H / luciferase transactivation assay / PCR
pPAL1r-HindIII	AT2G37040	GGATAAGCTTAACGTCCCCACCATAACACT	Cloning / Y1H / luciferase transactivation assay / PCR
qPAL1f	AT2G37040	GGAATATTCGGAAGCACGAA	qRT-PCR
qPAL1r	AT2G37040	CTCAAATCGGATACCGGAAA	qRT-PCR
pCYP71A12f-HindIII	AT2G30750	GGATAAGCTTTGGAAGTTTTATTTTAGTAATTAATCGTG	Cloning / Y1H / luciferase transactivation assay / PCR
pCYP71A12r-HindIII	AT2G30750	GGATAAGCTTTTGCATGAATGTAGCCCCTA	Cloning / Y1H / luciferase transactivation assay / PCR
qCYP71A12f	AT2G30750	GAGGCTTCCGTTGATTGGTA	qRT-PCR
qCYP71A12r	AT2G30750	ATGAGTGGTCCGTACCGAAG	qRT-PCR
pCYP71B15f-HindIII	AT3G26830	GGATAAGCTTTTTACTGACGGCTTCCTTTTT	Cloning / Y1H / luciferase transactivation assay / PCR
pCYP71B15r-HindIII	AT3G26830	GGATAAGCTTTCCGCTGTAGCTTGTGAATAA	Cloning / Y1H / luciferase transactivation assay / PCR
qCYP71B15f	AT3G26830	CGGAAGAATCGGTAGGTTCA	qRT-PCR
qCYP71B15r	AT3G26830	TCCAGGCTTAAGATGCTCGT	qRT-PCR
qEMB1144f	AT1G48850	CGTACCCAAACCAGGAAGAA	qRT-PCR
qEMB1144r	AT1G48850	ACCAACTCCTCCTCCATGTG	qRT-PCR
qF6'Hf	AT3G13610	AAACGGAGAGGAACCGATTT	qRT-PCR
qF6'Hr	AT3G13610	TCGACGGTTTTCTTTCCATC	qRT-PCR
qCAD5f	AT4G34230	GAATCTGCCACACCGATCTT	qRT-PCR
qCAD5r	AT4G34230	CATCTGATCCCACCTCCACT	qRT-PCR
qCOMTf	AT5G54160	TAGCCAGTGCTTCCGTTCTT	qRT-PCR
qCOMTr	AT5G54160	AGCGATCTCGGTAGGAGACA	qRT-PCR
qF5Hf	AT4G36220	ACCCGCAACATAACTTACCG	qRT-PCR
qF5Hr	AT4G36220	GAAATCCGCTACGTTGAAGG	qRT-PCR
qCYP79B2f	AT4G39950	CAACCGAAACATCGTCCTTT	qRT-PCR
qCYP79B2r	AT4G39950	TTGGGATCCGTCATCAATTT	qRT-PCR
qFOX1f	AT1G26380	AGCTGGCTTAACACGACGTT	qRT-PCR
qFOX1r	AT1G26380	GGGATTGGCTTTTTGACGTA	qRT-PCR
qCYP82C2f	AT4G3197	CCATCGAGAGGCGATAGAAG	qRT-PCR
qCYP82C2r	AT4G3197	ACCCTCGGATCTCTTTGGAT	qRT-PCR
qCYP71A13f	AT2G30770	TAGATGGGATCCGTGGTTTC	qRT-PCR
qCYP71A13r	AT2G30770	TCCACGAAATCCGCTTTATC	qRT-PCR
